# Addition of a Short Course of Prednisolone to a Gluten-Free Diet vs. Gluten-Free Diet Alone in Recovery of Celiac Disease: A Pilot Randomized Controlled Trial

**DOI:** 10.7759/cureus.2118

**Published:** 2018-01-28

**Authors:** Asad Abbas, Tabassum Shahab, Rana K Sherwani, Seema Alam

**Affiliations:** 1 District Early Intervention Centre, Aligarh Muslim University; 2 Jawaharlal Nehru Medical College, Aligarh Muslim University; 3 Department of Pathology, Aligarh Muslim University; 4 Department of Paediatric Hepatology, Institute of Liver and Biliary Sciences, New Delhi

**Keywords:** celiac disease, prednisolone, steroids, children, gluten-free diet (gfd)

## Abstract

Background

A gluten-free diet (GFD) is the standard of care in the management of patients with celiac disease, but clinical and histological recovery are often delayed. In newly diagnosed patients, strict compliance to GFD is difficult to achieve; this is especially true in developing countries where gluten-free food is often difficult to obtain. Steroids, when used alone, can be effective in inducing recovery in patients with celiac disease. We performed a randomized controlled trial to study the effect of a short course of prednisolone combined with a GFD on the recovery of celiac disease.

Materials and methods

This study was a single-center, randomised, open-label trial. This investigation was done in a pediatric gastroenterology unit of a tertiary teaching hospital in north India.Twenty-eight newly diagnosed celiac disease patients were enrolled in the study. Prednisolone was given at 1 mg/kg for four weeks; duodenal biopsies and IgA anti-tissue transglutaminase (tTg) levels were assessed at eight weeks, six months, and 12 months from the start of the study.

Outcome measures

The primary outcome measures used to indicate clinical, histological, and immunological recovery of celiac disease were clinical improvement at eight weeks and the proportion of patients with improved histology by at least one grade and who were tissue transglutaminase (tTg) seronegative at eight weeks. The secondary measures were the proportion of patients showing normalization of histological features and the proportions of patients becoming seronegative at six months and one year of GFD.

Results

Patients were randomized into the GFD only (n = 14) or GFD with prednisolone (GFD+P) (n = 14) groups. No significant differences were detected in clinical recovery at eight weeks; none of the patients became seronegative at eight weeks, six months, or 12 months. The proportion of patients with improvement in histology by at least one grade was higher in the GFD+P group at eight weeks, and there was no difference in overall histological improvement at 12 months after starting treatment.

Conclusion

The addition of a short course of prednisolone to a GFD does not affect clinical and serological recovery but might result in rapid histological recovery compared to a GFD alone in patients newly diagnosed with celiac disease.

## Introduction

It was established very early that patients with celiac disease respond either to a gluten-free diet (GFD) or corticosteroids. The efficacy of corticosteroids in the treatment of celiac disease was repeatedly demonstrated in newly diagnosed as well as refractory celiac disease [1-3]. Used alone, the effects of steroids were comparable to that of a GFD, and their absorption was not hampered, even in malabsorptive conditions like celiac disease [4-6]. The major problem with the use of oral steroids in celiac disease has been adrenal suppression. Steroids also lead to long-term side effects, such as osteoporosis, to which celiac disease increases vulnerability by hampering absorption of Vitamin D. Due to these reasons, steroids are currently reserved to treat celiac crisis or refractory disease [7-9]. However, short courses of steroids are relatively safe and have been used to treat refractory celiac disease and other inflammatory intestinal disorders, such as inflammatory bowel disease [10].

Currently, GFD is the favored method of managing celiac disease [11]. However, in newly diagnosed patients, strict compliance to GFD is difficult to achieve; this is especially true in developing countries where gluten-free food is often difficult to obtain [12]. Even on a strict GFD, histological recovery is often delayed and may never occur [13]. Clinical recovery also takes weeks to months, and even a small amount of gluten can cause relapse [14-15]. Many of the children are in the critical period of their growth where even weeks may matter and have a lasting impact on final growth. We hypothesized that the addition of a short course of steroids might have an adjunctive value in inducing early remission when used with a GFD by reducing immune-mediated destruction of villi. Therefore, we hypothesized that the addition of a short course of steroids to GFD might enhance intestinal mucosal recovery and thus possibly result in a faster clinical remission. We conducted a randomized control trial of the addition of a short course of steroids to a GFD in newly diagnosed celiac disease patients, and we evaluated subjects enrolled in the trial on the basis of improvement in symptoms at four weeks of starting treatment and improvement in histology by at least one grade and seronegativity at eight weeks of starting treatment. 

Aims

To assess the effects of the addition of steroids to GFD in patients with newly diagnosed celiac disease in terms of serological, histological and clinical improvement.

## Materials and methods

Subjects and settings

This randomized, open-label trial was conducted in departments of pediatrics and pathology in a tertiary teaching hospital in India. Clearance was obtained from the institute’s ethics committee, and informed consent was obtained from all individual participants, or their parents in the case of minors, included in the study. Patients presenting to pediatric outpatient clinic and gastroenterology clinic with signs and symptoms consistent with celiac disease were screened with immunoglobulin A (IgA) tissue transglutaminase (tTg) antibody assay and duodenal biopsies. The diagnosis of celiac disease was made based on the modified ESPGHAN criteria [16]. Newly diagnosed cases of celiac disease of both sexes in the age group of 1-18 years were randomly assigned to GFD only or GFD with prednisolone (GFD+P) group. We based study group assignment on a random number table, and personnel who were not involved in patient management placed the random numbers in opaque envelopes that were opened at the time of randomization. Subjects were enrolled for 18 months. Previously diagnosed cases of celiac disease who received GFD for any period were excluded. Other exclusion criteria were coexistent systemic disease, human immunodeficiency virus (HIV) seropositivity or hepatitis B surface antigen (HBsAg) positive, history of tuberculosis or evidence of active tuberculosis and patients who themselves or their parents were unwilling to participate in the study.

This study was also registered with the Clinical Trials Registry of India (registry number CTRI/2017/08/009517).

Study procedures

Upon randomization, the subjects assigned to GFD+P group were commenced on prednisolone (Wysolone, Wyeth, Madison, New Jersey, USA) at a dose of 1 mg/kg/day over a period of four weeks. GFD was given to both groups and was continued indefinitely. Patients in both groups were given information leaflets explaining about celiac disease and the foods items they could and could not eat. Nutritional supplementation in the form of iron, folate, vitamin B12, calcium, vitamin D, and multivitamins was received by both groups as recommended. We followed patients from each group weekly in the first month, fortnightly from one to three months, and every three months after that. Anthropometric measurements, as well as symptomatic improvement, were noted on each follow-up visit. A detailed dietary check history was taken, and adherence to GFD was reinforced. Safety of the study medication was assessed by monitoring the occurrence of any adverse event during the acute and follow-up phases of the study. In both groups, we conducted follow-up IgA anti-tTg antibody assay and duodenal biopsies after two months, six months, and one year of starting treatment. IgA anti-tTg antibody measurements were done by using a Thermo® iEMS™ microplate reader with an anti-tissue transglutaminase IgA ELISA kit (Thermo Scientific, Waltham, Massachusetts, USA). Duodenal biopsies were done by a physician trained in pediatric endoscopy. Tissue samples were taken from two to three sites. The tissue was put in formalin after orientation, and then the vial was labeled and sent immediately to a histopathology laboratory for further processing. In the histopathology lab, tissue was processed, assessing its size, amount, and color, and then was put in cassettes to be further processed. Sections were embedded in paraffin blocks, and from these, section slices of about 1 mm thickness were prepared by a manual tissue processing technique. The sections were then cut to 3-4 µm thickness with the help of a rotatory microtone and stained with hematoxylin and eosin. The slides were prepared and examined under 10X and 40X magnification. A histopathologist with interest in celiac disease examined the biopsy specimens without having any knowledge of the clinical profile of the patient. The modified Marsh grading system was used for grading mucosal changes [17].

We recorded symptoms and events that patients reported spontaneously, symptoms and events elicited in response to open-ended questions, and adverse effects observed at the follow-up visits. Each child was clinically evaluated for vital parameters, peripheral perfusion, weight, height, waist measurements, and assessed for infections. We performed measurements for blood sugar, hemoglobin, and serum proteins on each follow-up visit as well as electrolytes and a complete hemogram on selected patients if there was a need.

Study outcomes

The primary outcome measures used to indicate recovery from celiac disease were a clinical improvement in symptoms at eight weeks of starting treatment and the proportion of patients having improvement in histology by at least one grade and tTg seronegative at eight weeks of starting treatment. The secondary outcome measures were the proportion of patients showing normalization of histological features and the proportion of patients becoming seronegative at six months and one year of GFD. We took frequency of stools per day, hemoglobin level, abdominal circumference, weight for age, weight for height, total serum protein, and albumin as indicators of clinical improvement.

Statistical analysis

All statistical analyses were done on the intent-to-treat principle and included data from all randomized participants. All statistical assessments involved two-tailed tests and an alpha level of 0.05. Normally distributed continuous variables were expressed as mean values (standard deviation - SD). Categorical data were presented as proportions. Comparisons were done using χ^2^ test for discrete variables and the Mann-Whitney U test or t-test for continuous variables. The software package SPSS Statistics for Mac, version 17.0 (SPSS Inc., Chicago, IL, USA) was used for statistical analysis.

## Results

**Figure 1:**
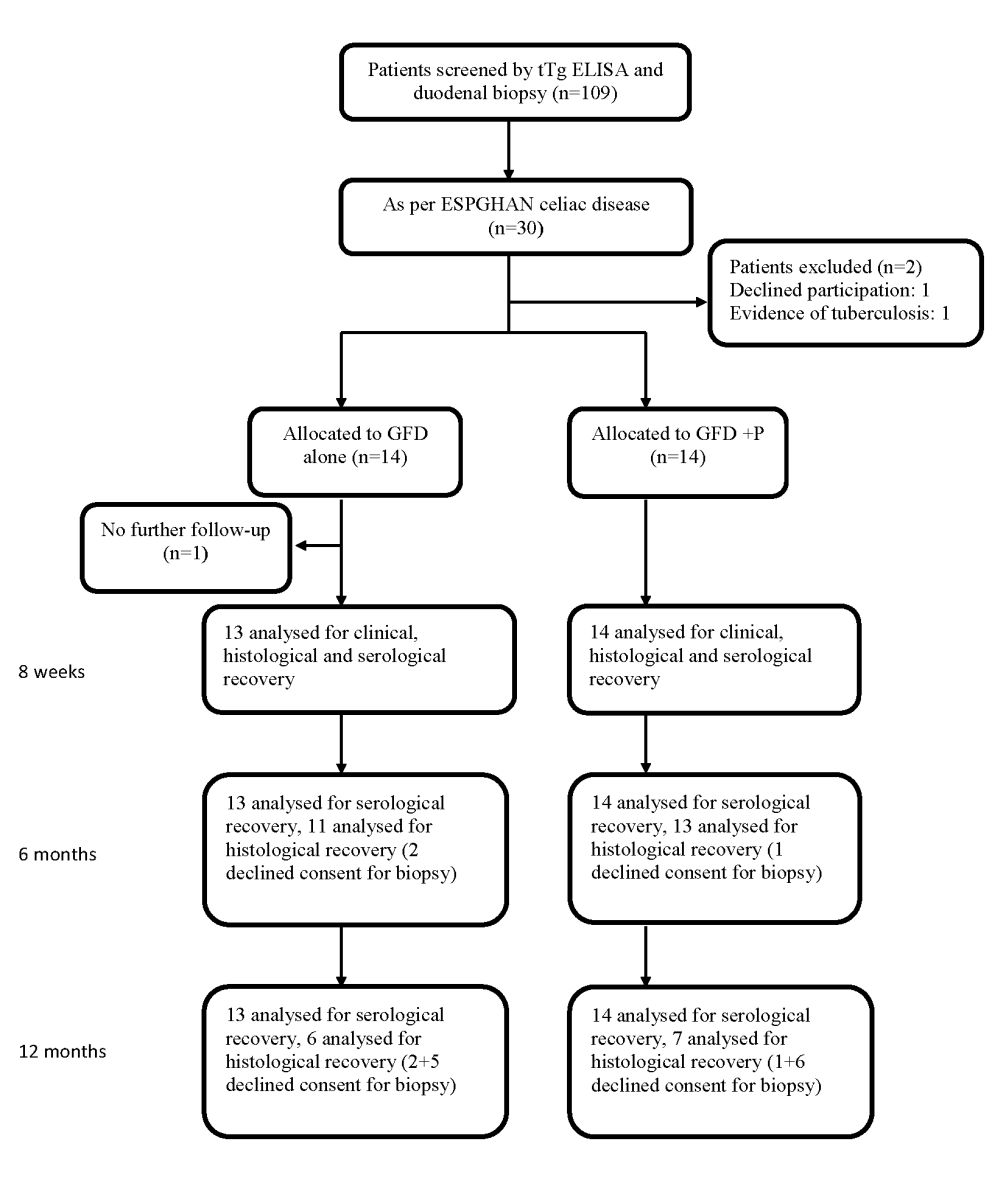
The flow of participants through each stage of this randomised trial. P, Prednisolone tTg: tissue transglutaminase; ELISA: enzyme-linked immunosorbent assay; n: number; ESPGHAN: European Society for Paediatric Gastroenterology, Hepatology and Nutrition; GFD: gluten-free diet; GFD+P: gluten-free diet, plus prednisolone

A total of 109 patients with signs or symptoms suggestive of classic celiac disease were screened. Thirty patients were diagnosed with celiac disease based on modified European Society for Paediatric Gastroenterology, Hepatology, and Nutrition (ESPGHAN) criteria. Two cases had to be excluded because of one or more exclusion criteria. Thus, a total of 28 patients were randomly assigned either to GFD only (n = 14) or GFP+P groups (n = 14). The number of patients who were screened and participated in the study are shown in Figure 1.

The two groups showed comparable baseline characteristics at admission (Table 1). Two patients from the GFD only group and one patient from GFD+P group declined consent for duodenal biopsy at six months and had no further biopsies, while five more patients from the GFD only group and six patients from the GFD+P group did not give consent for duodenal biopsy at 12 months. They, however, agreed to all other follow-ups. 

**Table 1:** Characteristics of the Study Population All values are represented as mean (standard deviation). GFD: gluten-free diet; GFD+P: gluten-free diet, plus prednisolone; n: number; tTg: tissue gransglutaminase

	GFD only group (n=14)	GFD+P group (n=14)	P value
Age (months)	89.5 (24.6)	76 (48.6)	0.36
Females, n (%)	4 (28.5)	5 (35.7)	0.68
Weight (kg)	16.4 (5.3)	16.5 (5.9)	0.96
Height (cm)	105.8 (14.1)	99.7 (22.0)	0.39
Weight for age (%)	66.2 (13.9)	70.2 (12.7)	0.43
Weight for height (%)	89.0 (14.3)	92.7 (11.3)	0.45
Hemoglobin (g/dL)	7.4 (2.0)	7.6 (2.7)	0.83
Number of stools per day (n)	6.6 (2.5)	7.8 (2.6)	0.22
Abdominal circumference (cm)	50 (8.9)	51.6 (7.3)	0.60
tTg value (IU/mL)	214.6 (116.3)	222.3 (103.2)	0.85
Marsh Score			
Marsh I	0	0	
Marsh II	0	0	
Marsh IIIA	0	1	
Marsh III B	1	0	
Marsh III C	12	11	
Marsh IV	0	1	

Table 2 shows the subjects’ primary outcomes. No clear differences in clinical parameters were found between the GFD only and GFD+P groups. The proportion of patients showing histological improvement by at least one Marsh grade was higher in GFD+P group, though the difference was not statistically significant. None of the patients in either group became seronegative, and there was no difference in the fall in anti-tTg antibody levels between the two groups.

**Table 2:** Primary Outcomes All values are represented as mean ± standard deviation, where appropriate. GFD: gluten-free diet; GFD+P: gluten-free diet, plus prednisolone; n: number; tTg: tissue gransglutaminase

Parameter	Baseline		8 weeks		P value
	GFD	GFD+P	GFD	GFD+P	
Abdominal circumference (cm)	50.0 ± 8.9	51.6 ± 7.3	43.7 ± 2.6	45.7 ± 1.7	0.41
Number of stools per day (n)	6.6 ± 2.5	7.8 ± 2.6	2.0 ± 0.75	2.4 ± 1.1	0.22
Weight for age (%)	66.2 ± 13.9	70.2 ± 12.7	73.1 ± 9.7	79.8 ± 10.5	0.90
Weight for height (%)	89.0 ± 14.3	92.7 ± 11.3	97.6 ± 13.9	104.9 ± 13.4	0.70
Hemoglobin (g/dL)	7.4 ± 2.0	7.6 ± 2.7	9.2 ± 1.7	10.1 ± 2.0	0.35
Total serum protein (g/dL)	6.9 ± 0.9	7.0 ± 0.5	7.3 ± 0.7	7.4 ± 0.6	0.35
Serum albumin (g/dL)	3.9 ± 0.4	3.8 ± 0.3	4.4 ± 0.6	4.5 ± 0.5	0.12
Histological improvement by at least one grade (n/N)	-	-	5/13	10/14	0.08
tTg seronegative (n)			0	0	
tTg value	214.6 ± 116.3	222.3 ± 103.2	95.6 ± 82.7	106.8 ± 53.0	0.11

As shown in Table 3, none of the patients from either group showed complete normalization of histology. The median improvement in the Marsh histological grade was higher by one grade in GFD+P group at six months. However, this difference was not seen at 12 months. Patients in both groups continued to have detectable anti-tTg antibodies at six and 12 months, and there was no difference between anti-tTg antibody levels between the two groups.

**Table 3:** Secondary Outcomes All values are represented as mean ± standard deviation, where appropriate. GFD: gluten-free diet; GFD+P: gluten-free diet, plus prednisolone; n: number; tTg: tissue gransglutaminase

	6 months		12 months	
	GFD	GFD+P	GFD	GFD+P
Normalization of histological features (n)	0	0	0	0
Median improvement in histological grade (interquartile range)	2 (1-3)	3 (1-4)	3 (1-3)	3 (1-3)
Improvement in histology (n)				
One grade	2	1	1	1
Two grades	4	3	2	0
Three grades	5	6	3	4
Four grades	0	1	0	1
tTg Seronegative	0	0	0	0
tTg value	53.1 ± 45.0	42.7 ± 25.0	39.3 ± 28.9	45.3 ± 25.2

No significant adverse effects were observed. One patient had mild hypertension, and two patients had hyperglycemia in GFD+P group; both normalized after stopping the drug.

## Discussion

Clinical recovery

In all the children studied, no significant differences were found between the clinical or laboratory parameters of the GFD only group and GFD+P group at eight weeks after starting treatment. When used alone, the efficacy of different steroids in the recovery from celiac disease has repeatedly been demonstrated. Lepore et al. demonstrated significant clinical improvement in six celiac disease patients after treatment with hydrocortisone at 40 mg/day for up to seven years while they continued a normal diet [4]. Bramble et al. observed the clinical response in five of 10 patients treated with betamethasone or clobetasone [2]. A study by Mitchison et al. used another topically acting corticosteroid, fluticasone propionate, at 20 mg/day for six weeks; this demonstrated clinical remission in seven patients and significant improvement in the rest of the five as evidenced by significantly improved hemoglobin concentration, decreased frequency of loose stools, and weight gain while continuing on a normal diet [3]. In studies where GFD was used along with steroids, Ciacci et al. demonstrated a better clinical response with a topically acting steroid, budesonide, and GFD, compared to GFD alone in 20 patients [18]. Shalimar et al., however, found no differences in clinical recovery while using prednisolone, 1 mg/kg for four weeks, along with GFD compared to GFD alone [19]. The findings in our study were consistent with those seen in the study by Shalimar et al. The lack of difference in clinical response might be explained by relatively shorter duration of steroid use in these studies. 

Histological recovery

The proportion of patients showing improvement in histological features by at least one grade was higher in the GFD+P group compared to the GFD only group, but the results were not statistically significant. The median improvement in histological grade was higher in the GFD+P group at six months; however, by 12 months, the median histological change was same in both groups. Wall et al. demonstrated improved cell height, villous recovery, and a reduction in lymphocytic infiltrates after treatment with prednisolone at 3 g/day for four to five weeks [1]. Radlovic et al. treated two infants with refractory celiac disease with a short course of prednisolone with good recovery and postulated that prednisolone may induce epithelial recovery [20]. Shalimar et al. demonstrated a rapid reduction in apoptotic activity when prednisolone was added to GFD. They also found, however, that prednisolone slowed down villous regeneration and concluded that a short course of prednisolone may be beneficial in patients in whom early histological improvement is required [19]. In this study, although the number of patients showing improvement in histology was higher in the GFD+P group, this effect was not maintained over a period of one year. This is consistent with the findings of the study by Shalimar et al. A short course of steroids might exert an anti-inflammatory effect in the short term; however, in the long term, the effects are negated by its inhibitory effects on villous regeneration. 

Serological recovery

None of the patients in either group became seronegative for tTg antibodies at eight weeks, and there were no differences in tTg levels between the two groups. Our study demonstrated a persistence of IgA anti-tTg antibodies at low levels, even at six to 12 months after starting GFD despite reported good compliance and complete clinical recovery. This might be attributable to some barriers to maintaining strict GFD in low- and lower/middle-income countries. Rajpoot et al. identified contamination of food during manufacturing, poor labeling of food packages with inadequate information about the disease, and lack of support from a nutritionist and celiac support groups as some of the factors that might lead to continued intake of small amounts of gluten [21]. Also, this study took place in a North Indian region where wheat is the staple food and its complete exclusion from the diet of the single family member is very difficult.

Our study had several limitations. Firstly, the number of participants in this study were small. Due to the same reason, we could analyze only a few of the more prevalent clinical parameters because the number of rarer clinical features was too small to include in the statistical analysis. Also, some parents from each group declined consent for duodenal biopsies once they felt their child’s clinical symptoms were improved. For these reasons, it is difficult to attribute the beneficial trend on histology seen to the use of prednisolone with GFD. Secondly, the criteria used to assess histological recovery was subjective and could have inter and intra-observer variations. A more stringent criterion using objective markers might give more reliable results. Thirdly, we did not use a placebo in our study. The finding of persistent anti-tTg antibodies might not be valid outside the geographic regions where wheat is not a staple. The strength of this study was its prospective nature and randomized design. The methods used for blinding and allocation concealment were robust, and we were able to retain most patients until the end of the study period. Prospective studies with a larger sample size with pre-specified sub-group analysis are needed to explore further the role of the addition of a short course of steroids in the recovery of celiac disease.

## Conclusions

The addition of a short course of prednisolone to GFD did not affect clinical and serological recovery but might result in faster histological recovery compared to GFD alone in patients newly diagnosed with celiac disease. A short course of steroids is safe, but whether its addition will result in significant benefits to patients newly diagnosed with celiac disease is not clear. A future study with larger sample size and more objective outcome measures is required to confirm these findings. 
